# High-resolution structures of the bound effectors avadomide (CC-122) and iberdomide (CC-220) highlight advantages and limitations of the MsCI4 soaking system

**DOI:** 10.1107/S2059798322000092

**Published:** 2022-02-18

**Authors:** Christopher Heim, Marcus D. Hartmann

**Affiliations:** a Max Planck Institute for Developmental Biology, Max-Planck-Ring 5, 72076 Tübingen, Germany; b Interfaculty Institute of Biochemistry, University of Tübingen, 72076 Tübingen, Germany

**Keywords:** cereblon, immunomodulatory imide drugs, avadomide, iberdomide, ubiquitin ligases, protein degradation, crystal soaking

## Abstract

Cereblon (CRBN) is the substrate receptor of the CRL4^CRBN^ E3 ubiquitin ligase and is a central player in targeted protein degradation. It is the target of the thalidomide-derived immunomodulatory drugs (IMiDs) and is one of the most widely employed receptors for proteolysis-targeting chimeras (PROTACs), both of which induce the ubiquitination and subsequent proteasomal degradation of target proteins. Structural studies of ligand binding to CRBN are crucial to elucidate the mechanisms of action and for mediation of side effects, ultimately aiding the development of next-generation IMiDs and PROTACs. With this aim, a crystal-soaking system based on the single-domain bacterial homologue MsCI4 has previously been established and used to delineate the binding modes of several classes of small molecules, including FDA-approved drugs, at the molecular level. Here, this system was used to characterize the binding of the next-generation IMiDs avadomide (CC-122) and iberdomide (CC-220) at high resolution, highlighting the advantages and limitations of the MsCI4 system and its implications for the development of future cereblon effectors.

## Abstract 2

Using the MsCI4 soaking system, the binding of the next-generation thalidomide-derived immunomodulatory drugs avadomide (CC-122) and iberdomide (CC-220) to cereblon was characterized at high resolution, highlighting the utility of the MsCI4 system for studies of the structure–activity relationship of cereblon effectors.

## 1. Introduction

For decades, thalidomide-derived immunomodulatory drugs (IMiDs) have been indispensable in the treatment of various diseases such as erythema nodosum leprosum (Walker *et al.*, 2007 ▸), myelodysplastic syndromes (Kale & List, 2006 ▸; Bertolini *et al.*, 2001 ▸) and most notably various forms of multiple myeloma (MM; Rajkumar *et al.*, 2005 ▸; Bjorklund *et al.*, 2020 ▸; Cruz, 2016 ▸; Krönke *et al.*, 2014 ▸). Their molecular targets and mechanisms of action remained elusive until the identification of cereblon (CRBN) as the primary target of thalidomide in 2010 (Ito *et al.*, 2010 ▸). CRBN serves as the substrate receptor of the CUL4–RBX1–DDB1–CRBN (CRL4^CRBN^) E3 ubiquitin ligase complex, where it is responsible for the recognition of endogenous substrates, including glutamine synthetase (Nguyen *et al.*, 2016 ▸, 2017 ▸) and MEIS2 (Fischer *et al.*, 2014 ▸). However, the binding of IMiDs changes the substrate specificity to target non-endogenous substrates, which are referred to as neo-substrates. Virtually all current IMiDs rely on the same glutarimide moiety for binding to CRBN, while the remainder of the molecule is unique to each IMiD. These unique moieties protrude into the solvent upon binding to CRBN, thereby mediating different neo-substrate specificities, which accounts for most of the efficacy of IMiDs.

The discovery that IMiDs bind to CRBN led to the rapid development of thalidomide-based proteolysis-targeting chimeras (PROTACs). In these, a binding moiety for a target protein is linked to a ligand for an E3 ligase, which was based on thalidomide in the first generations of CRBN-directed PROTACs; the first such PROTAC was reported by the Crews laboratory (Lu *et al.*, 2015 ▸), targeting BRD4. A multitude of CRBN-directed PROTACs are now available, including PROTACs targeting other E3 ligases (Steinebach, Kehm *et al.*, 2019 ▸; Girardini *et al.*, 2019 ▸). Further, convenient toolboxes for the synthesis of CRBN-directed PROTACs have been published (Steinebach, Sosič *et al.*, 2019 ▸) and chemical approaches for integrating IMiDs into PROTACs have been systematically studied (Bricelj *et al.*, 2021 ▸).

The rational development of newer-generation IMiDs and CRBN-directed PROTACs is critically dependent on structural information, and many laboratories have worked on the structural characterization of CRBN variants from human (Chamberlain *et al.*, 2014 ▸; Matyskiela *et al.*, 2016 ▸, 2018 ▸, 2020 ▸), mouse (Chamberlain *et al.*, 2014 ▸; Mori *et al.*, 2018 ▸), chicken (Fischer *et al.*, 2014 ▸) and bacteria (Lupas *et al.*, 2015 ▸; Hartmann *et al.*, 2014 ▸). In our laboratory, we established the single-domain bacterial homologue cereblon isoform 4 from *Magnetospirillum gryphiswaldense* (MsCI4) for structural ligand-binding studies, which shares 35% sequence identity and <1 Å r.m.s.d. over 100 C^α^ atoms with the thalidomide-binding domain (TBD) of human CRBN (Hartmann *et al.*, 2014 ▸, 2015 ▸). MsCI4 enabled us to establish biophysical assays, including a FRET assay (Boichenko *et al.*, 2016 ▸) and a microscale thermophoresis (MST)-based assay (Maiwald *et al.*, 2021 ▸; Heim *et al.*, 2021 ▸), for affinity determination, as well as a crystallographic soaking system. We grow MsCI4 crystals in complex with thalidomide, and then exchange thalidomide for the ligand in question in subsequent soaking experiments (Boichenko *et al.*, 2018 ▸; Heim *et al.*, 2019 ▸). This allows us to routinely determine the binding modes of CRBN effectors by X-ray crystallography at high resolution (<2 Å), which is currently not achievable for crystal structures of the human full-length protein. Using this soaking system, we have previously characterized the structural binding modes of many small molecules and FDA-approved drugs, such as aminoglutethimide, ethosuximide and rolipram (Boichenko *et al.*, 2018 ▸; Heim *et al.*, 2019 ▸). Here, we present the structural characterization of the next-generation IMiDs avadomide (CC-122) and iberdomide (CC-220), both of which are currently in clinical trials (Bristol-Myers Squibb & Celgene, 2021 ▸; Celgene, 2017 ▸, 2021*a* ▸), at resolutions of 1.52 and 1.80 Å, respectively, which highlight the advantages, but also the limitations, of the MsCI4 soaking system for structure–activity relationship (SAR) studies of CRBN effectors.

## 2. Materials and methods

### 2.1. Cloning, expression and purification of MsCI4

Wild-type MsCI4 was cloned, expressed and purified as described previously (Boichenko *et al.*, 2018 ▸; Heim *et al.*, 2019 ▸). In summary, MsCI4 was cloned into pETHis1a and used to transform *Escherichia coli* C41(DE3) cells. Cultures were grown in LB medium and protein expression was induced in the logarithmic phase. The cells were harvested and pelleted, resuspended in lysis buffer and sonicated. After centrifugation, the supernatant was subjected to Ni–NTA agarose separation before dialysis and TEV-catalyzed hydrolysis overnight to cleave the histidine tag. The mixture was loaded onto an Ni–NTA column, separating the cleaved MsCI4, which was concentrated to 17 mg ml^−1^ and stored at −80°C.

### 2.2. Labelled MST assay

To determine the affinity of the compounds for MsCI4, we covalently linked a fluorescent dye to the protein via amine-reactive cross-linking using Protein Labeling Kit RED-NHS 2nd Generation (Nanotemper Technology) according to the manufacturer’s protocol with a few optimizations. Buffer exchange into labelling buffer was performed using 30 µ*M* protein and it was diluted to 10 µ*M* for labelling. TCEP was included in the buffer at a final concentration of 0.1 m*M* in order to minimize oxidation of the protein. Fractions were collected after SEC column chromatography and the concentration/degree of labelling was measured using a spectrophotometer (NP-80, Implen). Aliquots were flash-frozen in liquid nitrogen and diluted 1:100 in MST buffer before measurements, which resulted in assay concentrations of ∼20 n*M*. Evaluation of the binding data was performed by plotting concentrations against the difference in MST behaviour (Δ*F*_norm_, ‰). Nonlinear regressions were calculated using the variable-slope model (4PL) integrated into *Prism* (GraphPad). Data were calculated using asymmetrical (profile-likelihood) models at a confidence level of 95%. Data are reported as the mean ± the standard error of the mean.

### 2.3. Protein crystallization and ligand soaking

**Figure 1:**
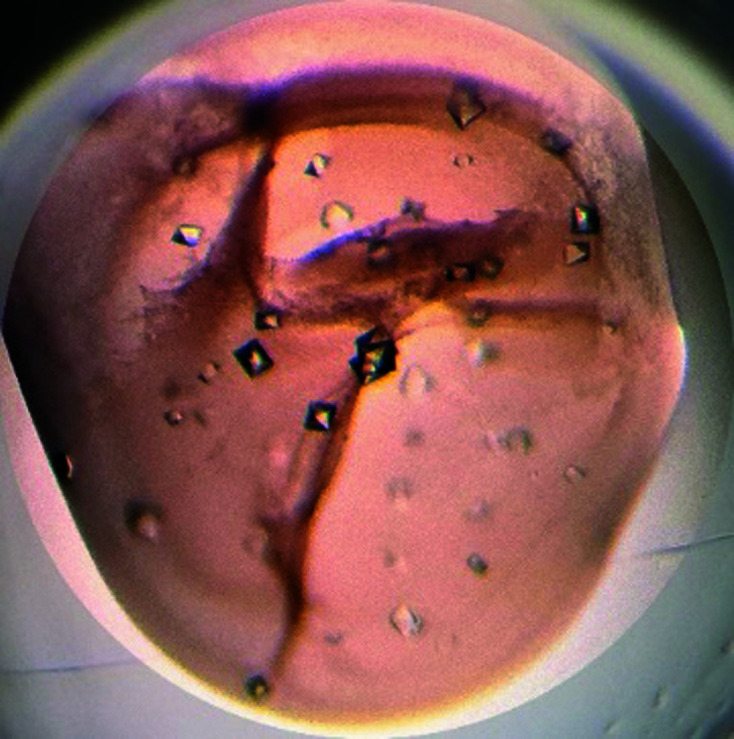
Crystals grown using the conditions given in Table 1 ▸.

As previously described, MsCI4 is highly amenable to crystallization, with crystals reliably growing from different conditions at 293 K (Boichenko *et al.*, 2018 ▸; Heim *et al.*, 2019 ▸). The crystals used in the soaking system can be obtained from two different crystallization conditions: one using PEG and the other using (NH_4_)H_2_PO_4_ at conentrations between 0.4 and 0.6 *M* as the sole precipitant. In both cases, the protein solution has to be concentrated to about 17 mg ml^−1^ in the presence of 3 m*M* thalidomide. Here, we were using crystals grown from the ammonium phosphate condition, which grew within a few days (Fig. 1 ▸).

For soaking trials, several crystals were transferred into 3 µl droplets of fresh reservoir solution. Compounds to be studied were then spiked into these drops by either adding 0.1 µl from a 20–100 m*M* stock solution in DMSO or small amounts of dried compound. To fully exchange thalidomide for the ligand in question, soaking times of greater than 24 h are required. Further treatment to remove thalidomide is not required, but it is possible to ‘wash’ thalidomide out of its binding pocket by incubating crystals in droplets without ligand for about 40 h (Hartmann *et al.*, 2015 ▸). In this study, soaking drops with avadomide and iberdomide were covered with a lid to prevent them drying out and were incubated for up to 72 h at 293 K. Crystals were cryoprotected via transfer into fresh reservoir solution supplemented with 70% sodium malonate for 30 s and were flash-cooled in liquid nitrogen. Crystallization information is summarized in Table 1 ▸.

### 2.4. Data collection, processing and structure determination

Diffraction data were collected at 100 K on beamline X10SA at the Swiss Light Source using a PILATUS 6M-F hybrid pixel detector (Dectris). The data were processed and scaled using *XDS* (Kabsch, 2010 ▸) and the structures were solved by difference Fourier methods using PDB entry 4v2y as the starting model and were completed via refinement with *REFMAC*5 (Murshudov *et al.*, 2011 ▸) and cyclic modelling using *Coot* (Emsley *et al.*, 2010 ▸). Molecular figures were generated using *PyMOL* (version 2.5.0; Schrödinger). Data-collection and processing statistics are summarized in Table 2 ▸, and structure solution and refinement statistics in Table 3 ▸. The structures were deposited in the Protein Data Bank (PDB) with accession codes 7pso and 7ps9.

## 3. Results and discussion

### 3.1. Determination of affinities for MsCI4 using a labelled MST assay

**Figure 2:**
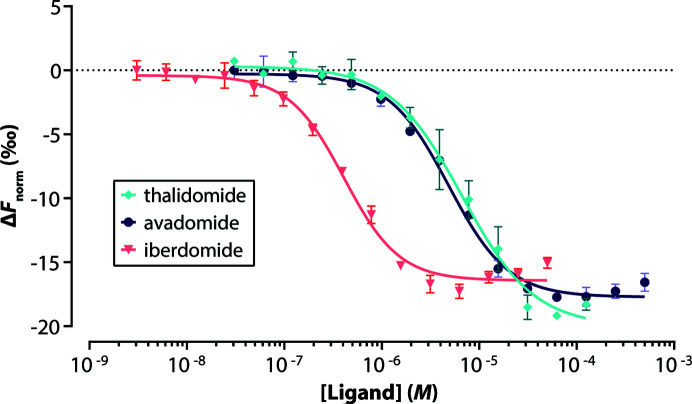
Dose–response curves for determination of *K*_d_ for the binding of IMiDs to MsCI4 using a labelled MST assay.

In our previously reported custom assays for the characterization of CRBN ligands, avadomide and iberdomide behaved problematically for different reasons. While in the MsCI4-based FRET assay these compounds were intractable due to their inherent autofluorescence, they were tractable for human TBD (hTBD) but not for the MsCI4 protein in the competitive MST assay (Boichenko *et al.*, 2016 ▸; Maiwald *et al.*, 2021 ▸). To allow comparison of their binding affinities to MsCI4 and hTBD, we resorted to a labelled MST assay, in which we follow ligand binding to fluorescently labelled MsCI4 (Fig. 2 ▸, Table 4 ▸). These data showed a clear correlation between the different assays and protein constructs. Compared with thalidomide, avadomide showed a slightly increased affinity, while a more than tenfold gain in affinity was observed for iberdomide.

### 3.2. Ligand binding in the MsCI4 soaking system

**Figure 3:**
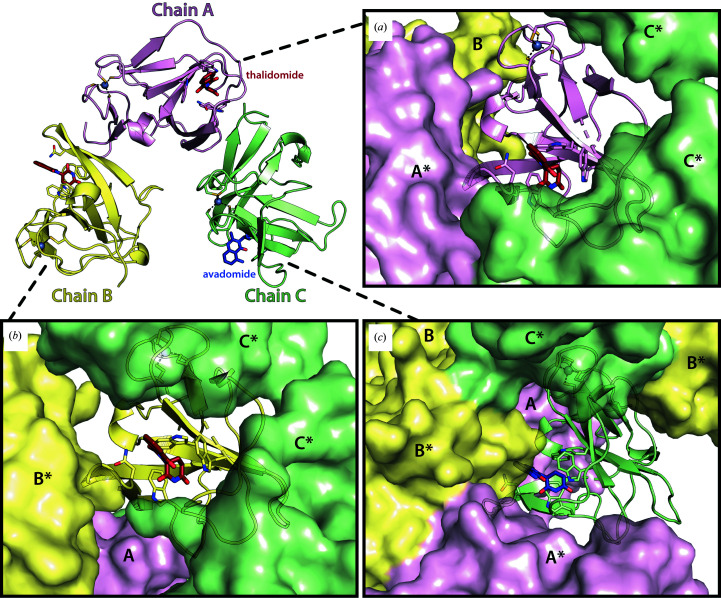
Overview of the asymmetric unit and crystal packing in the MsCI4 crystals used for soaking. (*a*, *b*, *c*) Views of the bound ligands in the binding pocket (PDB entry 7pso, this study) of the three different chains (shown as a cartoon), illustrating their unique environment due to neighbouring chains (shown as a surface). An asterisk indicates a symmetry-related chain.

**Figure 4:**
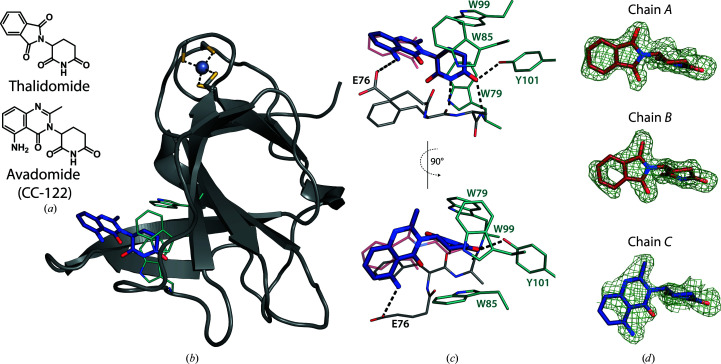
Binding of avadomide to MsCI4. (*a*) Chemical drawings of thalidomide and avadomide. (*b*) Cartoon representation of MsCI4 in complex with avadomide, highlighting the tryptophans of the aromatic cage and the structural zinc ion. (*c*) Interactions within the binding pocket, with the binding pose of thalidomide superposed in purple (PDB entry 4v2y), highlighting the additional interaction of the amine with Glu76. (*d*) *F*_o_ − *F*_c_ omit maps of thalidomide (chains *A* and *B*) and avadomide (chain *C*) at a contour level of 1.5σ. As generally observed for the phthaloyl moiety of thalidomide, the distal parts of the protruding quinazolinone moiety are less defined in the electron density.

The crystals employed in the soaking system belong to the orthorhombic space group *P*2_1_2_1_2_1_ and contain three monomers in the asymmetric unit, each engaging in different crystal contacts (Fig. 3 ▸). The most relevant region of each MsCI4 monomer is the thalidomide-binding pocket, which is solvent-accessible in all three monomers. This pocket is formed by the three conserved tryptophan residues Trp79, Trp85 and Trp99, which correspond to Trp380, Trp386 and Trp400 in the human protein. Compounds based on glutarimide bind to this pocket with the following interactions. The glutarimide ring forms two canonical hydrogen bonds: one between the amino group and the backbone of Phe77 (His378 in hCRBN) and the other between the distal keto group and the backbone of Trp79 (Trp380 in hCRBN) (Fig. 4 ▸). A third hydrogen bond is specific to organisms that carry a Phe→Tyr substitution at the base of the aromatic cage, as is the case in MsCI4. The distal keto group of glutarimide forms this additional bond with the hydroxyl group of Tyr101, which is absent in the human protein; using an MsCI4^Y101F^ mutant construct (Hartmann *et al.*, 2014 ▸; Boichenko *et al.*, 2016 ▸) we previously verified that this additional bond increases the affinity but does not alter the binding modes of small molecules. In addition to high-affinity ligands, this system was successfully used to study low-affinity binders (>100 µ*M*) such as γ-valerolactam and rolipram (Boichenko *et al.*, 2018 ▸; Maiwald *et al.*, 2021 ▸). Empirically, we saw that during the soaking experiments different molecules can selectively bind to individual chains of the asymmetric unit, with some effectors replacing thalidomide in all three chains (Boichenko *et al.*, 2018 ▸; Heim *et al.*, 2019 ▸). In this study, we observe that avadomide is clearly bound to chain *C*, with remaining thalidomide molecules in chain *A* and *B*, whereas iberdomide is clearly present in all three monomers.

### 3.3. Structure of MsCI4 in complex with avadomide (CC-122)

Avadomide is a next-generation nonphthalimide analogue of thalidomide that is currently in Phase I clinical trials for advanced melanoma (Bristol-Myers Squibb, 2021 ▸) and in Phase II clinical trials for solid tumours and MM (Celgene, 2021*b* ▸). The IMiD consists of a glutarimide moiety and an aminomethyl quinazolinone, both of which were resolved in the electron density in chain *C*, as depicted in the respective *F*_o_ − *F*_c_ omit map in Fig. 4 ▸. As expected, the canonical binding mode was observed for the glutarimide moiety, showing virtually no difference to the binding of thalidomide (Fig. 4 ▸). Compared with the latter, the protruding moiety is slightly shifted upwards towards Trp79 due to the geometry of the quinazolinone, which is bulkier than the phthaloyl moiety in thalidomide. Moreover, it forms an additional interaction between its distal amino group and the conserved glutamic acid Glu76 (Glu377 in hCRBN), which is likely to contribute to the slight improvement in binding affinity over thalidomide in MsCI4 and hTBD (Table 4 ▸). However, the high potency reported for the degradation of neo-substrates, including Ikaros and Aiolos (Hagner *et al.*, 2015 ▸), is unlikely to be solely due to the slight gain in affinity to CRBN. Additional factors mediating this efficacy could include hydrophobic interactions of the bulkier quinazolinone and potential hydrogen bonds between its secondary amine group and the neo-substrates, as well as its pharmacological parameters.

### 3.4. Structure of MsCI4 in complex with iberdomide (CC-220)

**Figure 5:**
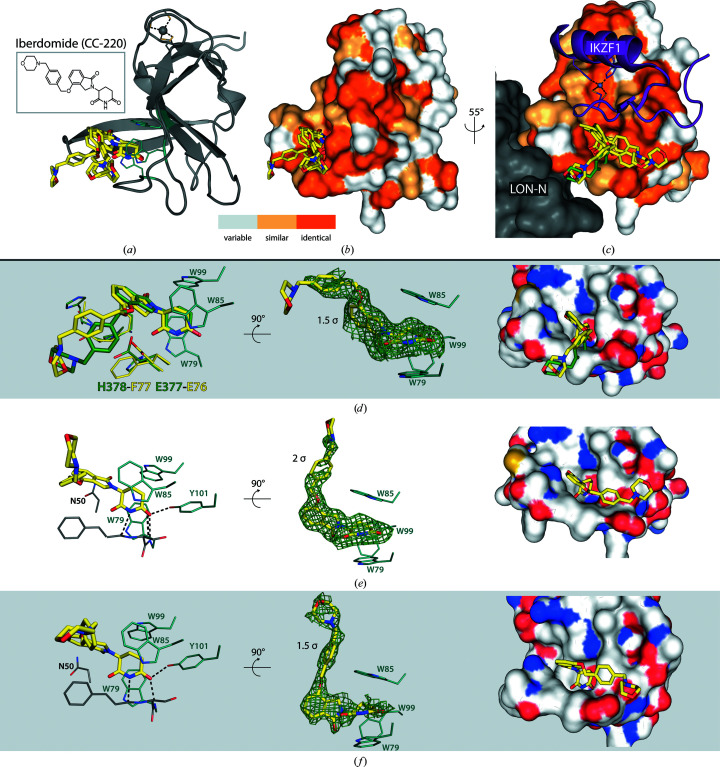
Binding of iberdomide to MsCI4. (*a*) Superposition of chains *A*, *B* and *C*, showing differences in the protruding moieties of iberdomide. (*b*) Coloured surface representation of MsCI4 showing the residue conservation between MsCI4 and hTBD, according to the sequence alignment depicted in Fig. 6 ▸. (*c*) Superposed structures of MsCI4–iberdomide, hCRBN–iberdomide (PDB entry 5v3o, iberdomide in green) and hCRBN–pomalidomide–IKZF1 (PDB entry 6h0f) showing the possible compatibility of alternative binding modes of iberdomide with the binding of the neo-substrate IKZF1. (*d*, *e*, *f*) Interactions of iberdomide within the different chains, with iberdomide from PDB entry 5v3o superposed in green. *F*_o_ − *F*_c_ omit maps are shown with the indicated contour levels. On the right, the binding poses of iberdomide are shown with the protein surface coloured according to amino-acid chemistry, coloured by atom type.

The IMiD iberdomide, which is about to enter Phase III clinical trials (Celgene, 2021*a* ▸) for different forms of MM, consists of an extended tail formed by a morpho­linomethyl-benzyl group connected via an oxy group to phthalimidine, which is attached to a classical glutarimide moiety. In our soaking trials, the prolonged effector bound to all three chains of MsCI4 with clearly defined electron density for most of the molecules (Fig. 5 ▸). Most interestingly, the orientation of the protruding moiety was different in all three chains. The ligand in chain *A* is oriented similarly to that in a previously reported human complex structure (PDB entry 5v3o; Matyskiela *et al.*, 2018 ▸). While the glutarimide moiety shows the canonical interactions with the tri-Trp pocket, the phenyl ring is harboured in a channel formed by Glu76, Phe77 and Pro51 of MsCI4. The human CRBN possesses an additional histidine, His353, which contributes to this groove. This corresponds to Ala52 in MsCI4, which cannot contribute to this interaction. Interestingly, the morpholine ring, which was modelled to be oriented towards a hydrophobic pocket formed by Ile154 and Phe102 in human CRBN, showed only poor electron density in both the human structure and in this structure, underpinning its conformational flexibility.

While the glutarimide moiety of iberdomide is bound in an identical fashion in chains *B* and *C*, the protruding moiety is found in a very different orientation, filling a surface groove behind Trp85. In chain *C*, the orientation of the molecule could partially be influenced by crystal contacts with Ile70, Gly71 and Ala72 of the symmetry chain *B*. However, the protruding moiety in chain *B* adopts a very similar orientation, albeit being solvent-exposed with the nearest symmetry atoms more than 7 Å away, suggesting that this overall conformation is not the result of crystal-packing effects.

Although the binding mode in chain *A* is similar overall to that observed for the co-crystal structure of the human protein, and the mode in chain *B* is similar overall to that in chain *C*, we are looking at four different binding modes. This leads to the question whether one binding mode is preferred over the others, and which one that might be. As MsCI4 is similar but not identical to hTBD, one has to consider the conservation of the surface areas that are in contact with the ligand. Interestingly, as depicted in Fig. 5 ▸, especially in chains *B* and *C*, in which the binding mode deviates the most from that observed in the crystal structure with the human protein, the ligand is mostly in contact with conserved surface areas. This suggests that iberdomide might be able to bind in a number of different conformations and that the one previously reported in hTBD, just like the three observed with MsCI4, may not be the only or the most relevant one(s).

As the ligand is found to collapse onto the protein surface in all four conformations, all are compatible with the greater than tenfold increase in binding affinity that we observe for MsCI4 and hTBD (Table 4 ▸). Further, as illustrated in the superposition in Fig. 5 ▸, all four are compatible with the domain architecture of the whole E3 ligase complex and all would allow the binding of neo-substrates such as the zinc fingers of Ikaros and Aiolos, as the compound is oriented away from the binding interface in all cases. This in turn raises the question of whether iberdomide would possibly adopt another different conformation in the ternary complex to also form additional interactions with the neo-substrate. It remains uncertain whether one of the poses reported here for iberdomide, or the previously reported pose, is the pose that mediates neo-substrate binding.

### 3.5. Approaches for the humanization of bacterial CRBN

**Figure 6:**
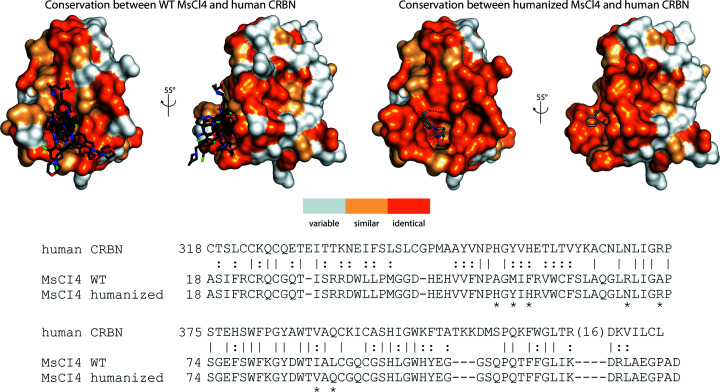
Sequence conservation of MsCI4 and human CRBN. Sequence conservation according to the sequence alignment of hTBD with wild-type (WT) MsCI4 and humanized MsCI4 is mapped onto the surface of MsCI4. Identical residues are shown in dark orange in the surface representation and marked with ‘|’ in the sequence alignment; similar residues are shown in light orange and highlighted with ‘:’. Mutations introduced in the humanized MsCI4 construct are marked with ‘*’. The superposition of effector-bound MsCI4 structures at the top left includes PDB entries 4v2y, 4v30, 4v31, 5oh1, 5oh3, 5oh8, 6r0s, 6r0v, 6r11, 6r12, 6r18, 6r19, 6r1a, 6r1c, 6r1d, 6r1k, 6r1w and 6r1x. The structure at the top right shows the previously published humanized MsCI4 construct in complex with the hydrolyzed thalidomide molecule α-(2-carboxybenzamido)glutarimide (PDB entry 6r0q).

MsCI4 has proven to be a reliable system for the characterization of cereblon binders. The aromatic cage is highly conserved across species, and the binding modes for classical IMiDs were found to be identical between the human and bacterial proteins. This allowed us to characterize many small molecules and FDA-approved drugs (Boichenko *et al.*, 2018 ▸; Maiwald *et al.*, 2021 ▸). Fig. 6 ▸ illustrates that all compounds studied in MsCI4 almost exclusively contact surface areas that are conserved between MsCI4 and hTBD. However, as the compounds become longer in order to improve their affinity and selectivity towards neo-substrates, they start protruding into areas on the surface where species-specific differences can become relevant and can potentially influence binding poses. In the absence of a high-resolution soaking system for the human protein, one possibility to circumvent the ambiguity arising from species-specific differences is to humanize the bacterial protein. A minimal example for this approach is the previously mentioned MsCI4^Y101F^ variant, which has a chemically identical aromatic cage. Furthermore, we previously created a humanized MsCI4 variant that selectively captured a hydrolyzed form of thalidomide (Fig. 6 ▸; Heim *et al.*, 2019 ▸). This exemplifies how further interactions with the human protein can be captured, and further surface mutations around the vicinity of the aromatic cage are conceivable to create an identical environment for elongated cereblon binders. Although we have successfully used this construct in co-crystallization trials with a number of compounds in addition to that depicted in Fig. 6 ▸, we have so far not been able to obtain a soakable system that could be employed as widely as the presented (wild-type) MsCI4 system.

## 4. Conclusions

In summary, our results clearly highlight the advantages, but also the limitations, of using MsCI4 as a soaking system. MsCI4 crystals can be grown reproducibly under a variety of conditions and generally diffract to high resolution, even after extensive soaking trials. To date, this soaking system has proven to be a reliable surrogate system to determine the binding of many small molecules, ranging from low-affinity to high-affinity binders, including immunomodulatory drugs, their analogues and hydrolysis products, and a variety of FDA-approved drugs, at high resolution. The solvent-accessible binding pockets of three subunits per asymmetric unit increase the chance of capturing the binding of effectors and potentially alternative binding modes in one crystal structure. In this way, we could show that avadomide is essentially bound in the canonical IMiD-binding mode with an additional hydrogen bond, whereas alternative binding modes were captured for the effector iberdomide. For such extended compounds, it cannot be excluded that species-specific amino acid differences between MsCI4 and hTBD lead to differences in the binding modes. It is obviously desirable to have a high-resolution soaking system based on hTBD, but as long as such a system is not available, we continue working on the establishment of such a system based on further humanized MsCI4 variants in parallel. We note that a soaking system, like any other biophysical *in vitro* technique, cannot capture all aspects of drug efficacy, as it does not consider general pharmaco­kinetic (ADME) parameters. However, there is also a general limitation of any soaking system for CRBN ligands to keep in mind. A soaking system is an excellent tool to study ligand binding to CRBN, which can be especially useful for the development of PROTAC building blocks. For IMiDs, however, it does not necessarily deliver solid insights into how ligand binding influences the binding of neo-substrates, as mirrored by the conformational ambiguity of the observed iberdomide binding modes. Consequently, in contrast to PROTACs, studying the modes of action of IMiDs will also require ternary complexes together with the respective neo-substrate, which are not generally achievable in a soaking system.

## Supplementary Material

PDB reference: cereblon isoform 4 from *Magnetospirillum gryphiswaldense*, complex with iberdomide (CC-220), 7ps9

PDB reference: complex with avadomide (CC-122), 7pso

## Figures and Tables

**Table 1:** Crystallization conditions

Method	Sitting drop
Plate type	Intelli-Plate 96-3
Temperature (K)	293
Protein concentration (mg ml^−1^)	17
Buffer composition of protein solution	20 m*M* Tris–HCl pH 7.5, 150 m*M* NaCl, 5 m*M* β-mercaptoethanol
Composition of reservoir solution	0.4–0.6 *M* (NH_4_)H_2_PO_4_
Volume and ratio of drop	400 nl, 1:1
Volume of reservoir (µl)	50

**Table 2:** Data collection and processing Values in parentheses are for the outer shell.

	MsCI4–avadomide	MsCI4–iberdomide
Diffraction source	X10SA, Swiss Light Source	X10SA, Swiss Light Source
Wavelength (Å)	1	1
Temperature (K)	100	100
Detector	PILATUS 6M-F	PILATUS 6M-F
Crystal-to-detector distance (mm)	220	250
Rotation range per image (°)	1	1
Total rotation range (°)	360	360
Exposure time per image (s)	0.1	0.1
Space group	*P*2_1_2_1_2_1_	*P*2_1_2_1_2_1_
*a*, *b*, *c* (Å)	56.56, 60.16, 88.41	56.00, 58.85, 88.58
α, β, γ (°)	90, 90, 90	90, 90, 90
Mosaicity (°)	0.224	0.321
Resolution range (Å)	44.24–1.52 (1.61–1.52)	34.74–1.80 (1.91–1.80)
Total No. of reflections	599868 (97126)	342611 (50061)
No. of unique reflections	47297 (7506)	27318 (4220)
Completeness (%)	99.9 (99.4)	97.7 (94.9)
CC_1/2_	0.998 (0.845)	0.999 (0.783)
Multiplicity	12.7 (12.9)	12.5 (11.9)
〈*I*/σ(*I*)〉	18.96 (1.54)	11.24 (1.21)
*R*_r.i.m._	0.069 (1.39)	0.14 (1.61)
Overall *B* factor from Wilson plot (Å^2^)	24.0	29.9
PDB code	7pso	7ps9

**Table 3:** Structure solution and refinement Values in parentheses are for the outer shell.

	MsCI4–avadomide	MsCI4–iberdomide
Resolution range (Å)	44.20–1.52 (1.56–1.52)	34.76–1.80 (1.86–1.80)
Completeness (%)	99.9	97.7
σ Cutoff	None	None
No. of reflections, working set	44930 (3246)	25945 (1777)
No. of reflections, test set	2367	1373
Final *R*_cryst_	0.18	0.19
Final *R*_free_	0.22	0.25
Cruickshank DPI	0.0817	0.1443
No. of non-H atoms		
Total	2739	2588
Protein	2481	2347
Ion	18	3
Ligand	59	99
Water	181	139
R.m.s. deviations		
Bond lengths (Å)	0.012	0.013
Angles (°)	1.862	1.730
Average *B* factors (Å^2^)		
Overall	31.3	40.3
Protein	30.9	39.2
Ion	56.2	30.8
Ligand	30.5	62.3
Water	40.6	43.2
Ramachandran plot		
Most favoured (%)	99.04	96.22
Allowed (%)	0.96	3.78

**Table 4:** Comparison of the binding affinities of IMiDs to MsCI4 and hTBD

Protein	MsCI4 (*K*_d_, µ*M*)	MsCI4 (*K*_i_, µ*M*)	hTBD (*K*_i_, µ*M*)
Assay	Labelled MST	FRET (Boichenko *et al.*, 2016 ▸)	Competitive MST (Maiwald *et al.*, 2022 ▸)
Thalidomide	6.68 ± 0.8	4.4	8.51 ± 0.83
Avadomide	4.84 ± 0.3	n.d.	6.66 ± 0.94
Iberdomide	0.409 ± 0.03	n.d.	0.765 ± 0.31
